# Near Infrared Quantum Cutting Luminescence of Er^3+^/Tm^3+^ Ion Pairs in a Telluride Glass

**DOI:** 10.1038/s41598-017-02244-8

**Published:** 2017-05-16

**Authors:** Xiaobo Chen, Song Li, Lili Hu, Kezhi Wang, Guoying Zhao, Lizhu He, Jinying Liu, Chunlei Yu, Jingfu Tao, Wei Lin, Guojian Yang, Gregory J. Salamo

**Affiliations:** 1 Applied Optics Beijing Area Major Laboratory and Physics Department, Beijing Key Laboratory of Energy Conversion and Storage Materials, Beijing Normal University, Beijing, 100875 China; 2 Shanghai Institute of Optics and Fine Mechanics, Chinese Academy of Science, Shanghai, 201800 China; 3 Research Center of Shanghai High Power Laser Glass, Chinese Academy of Science, Shanghai, 201800 China; 4 College of Chemistry, Beijing Normal University, Beijing, 100875 China; 5 School of Materials Science and Technology, Shanghai Institute of Technology, Shanghai, 200235 China; 6 School of Materials Science and Engineering, University of Science and Technology Beijing, Beijing, 100083 China; 7 Department of Physics, University of Arkansas, Fayetteville, AR 72701 USA

## Abstract

The multiphoton near-infrared, quantum cutting luminescence in Er^3+^/Tm^3+^ co-doped telluride glass was studied. We found that the near-infrared 1800-nm luminescence intensity of (A) Er^3+^(8%)Tm^3+^(0.5%):telluride glass was approximately 4.4 to 19.5 times larger than that of (B) Tm^3+^(0.5%):telluride glass, and approximately 5.0 times larger than that of (C) Er^3+^(0.5%):telluride glass. Additionally, the infrared excitation spectra of the 1800 nm luminescence, as well as the visible excitation spectra of the 522 nm and 652 nm luminescence, of (A) Er^3+^(8%)Tm^3+^(0.5%):telluride glass are very similar to those of Er^3+^ ions in (C) Er^3+^(0.5%):telluride glass, with respect to the shapes of their excitation spectral waveforms and peak wavelengths. Moreover, we found that there is a strong spectral overlap and energy transfer between the infrared luminescence of Er^3+^ donor ions and the infrared absorption of Tm^3+^ acceptor ions. The efficiency of this energy transfer {^4^I_13/2_(Er^3+^) → ^4^I_15/2_(Er^3+^), ^3^H_6_(Tm^3+^) → ^3^F_4_(Tm^3+^)} between the Er^3+^ and Tm^3+^ ions is approximately 69.8%. Therefore, we can conclude that the observed behaviour is an interesting multiphoton, near-infrared, quantum cutting luminescence phenomenon that occurs in novel Er^3+^-Tm^3+^ ion pairs. These findings are significant for the development of next-generation environmentally friendly germanium solar cells, and near-to-mid infrared (1.8–2.0 μm) lasers pumped by GaN light emitting diodes.

## Introduction

With the gradual depletion of fossil fuel energy sources and the increasing pollution of the environment, the development of new energy sources has become of utmost importance^1–12^. The most promising new energy source is solar energy. However, for current solar cells, the photoelectric transfer cost is high, and the efficiency is low. This results in a large difference between the significant potential of solar energy and its actual utilization rate^5–20^. Through quantum cutting, a high-energy photon can be converted into many low-energy photons. It is a new method to reduce the losses in solar cells by modifying the distribution of the incident solar light energy, which can be used to generate solar energy more effectively^5, 12–33^. It is possible to apply the quantum cutting method to all types of solar cells without changing their structures. The ability of photovoltaic cells to convert sunlight into electricity makes them prime candidates for the effective large-scale capture and conversion of solar energy.

Green and Trupke originally proposed the theory of a “two-photon quantum cutting silicon solar cell” in 2002^10^. They reported a maximum theoretical efficiency of 38% for such a device, and it exhibited sensitivity to solar light at wavelengths from 280 nm to 1100 nm^10^. Meijerink and Vergeer first demonstrated an experiment on the near-infrared, two-photon quantum cutting phenomenon in Yb_x_Y_1−x_PO_4_:Tb^3+^ phosphors in 2005^1^, which was conducted after they reported a well-known visible quantum cutting experiment for an Eu^3+^/Gd^3+^ system in *Science* ^2^. Since 2007, several groups, including Meijerink^1, 3, 19, 21, 27^, Qiu and Zhou^5, 12, 15^, Wang and Chen^6, 11, 13^, Huang^24^, Zou and Wang^25^, Fedorov and Luginina^9^, Zhang^7, 27^, Xia and Hu^29^, Guo and Chen^23^, and Song and Tao^30, 32^, and more^17, 20, 22^, have published more than 200 articles on the second-order, near-infrared, quantum cutting luminescence phenomena of sensitizer-Yb^3+^ co-doped materials^1–25^, which were used to develop two-photon quantum cutting silicon solar cells. Near-infrared quantum cutting has become a hot topic in the field of science and nature. Recently, it was proven by several groups, including Hu and Hao^22^, Li^17^, and Song^33^, etc. that the actual photoelectric conversion efficiency of silicon based solar cells can be enhanced by the quantum cutting effect. Meijerink^19, 27^, Qiu and Zhou^12, 15^, Zhang^7, 27^, Huang^24^, and our group^8, 28^ have reported experimental research on first-order, multiphoton, near-infrared quantum cutting in Er^3+^ or Tm^3+^ activator-ion doped materials. This improvement has led to the development of first-order, multiphoton quantum cutting germanium (Ge) and silicon–germanium (Si–Ge) solar cells^1, 7, 8, 12, 15, 27, 28^, which are sensitive to wavelengths of 280–1850 nm and are environmentally friendly. Their maximum efficiency can clearly exceed 38%. On the other hand, near-infrared quantum cutting can be used in 1.8–2.0 μm near-to-mid infrared lasers. These types of lasers have potential applications in micro-surgery, tissue welding, range finding, remote sensing, environmental trace-gas detection, and biophysical applications^12, 31^.

One key factor has contributed to the important developments and improvements of first-order near-infrared quantum cutting, compared to second-order methods. Because there are no energy resonances between donors and acceptors and its energy of a donor equals two times the energy of an acceptor for second-order near-infrared quantum cutting, thus it only has a second-order process but not a first-order process. However, a first-order process is approximately 1000 times larger than a second-order process, as indicated by Meijerink^3^. In particular, for the first-order near-infrared multiphoton quantum cutting of Er^3+^ or Tm^3+^ ions, their cross energy transfers are excellent first-order processes with high oscillator intensities and small energy mismatches. Therefore, the first-order, near-infrared, multiphoton quantum cutting processes with Er^3+^ or Tm^3+^ activators have large rates, high efficiencies, and excellent prospects for different applications.

## Results

### Absorption

**Figure 1:**
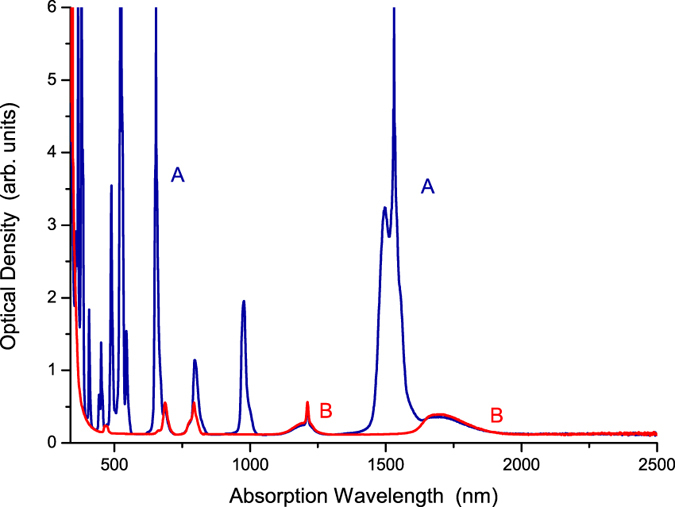
Absorption spectra of samples (A) Er^3+^(8%)Tm^3+^(0.5%):telluride glass and (B) Tm^3+^(0.5%):telluride glass.

**Figure 2:**
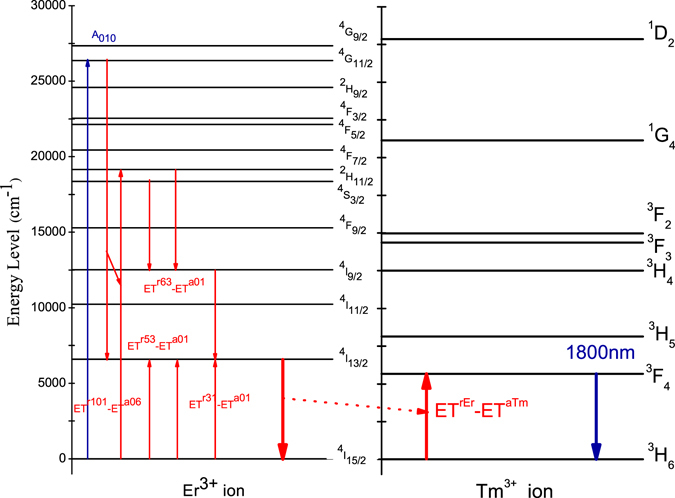
Schematic diagrams of the energy-level structures of Er^3+^ and Tm^3+^ ions and the quantum cutting process.

The absorption spectra of samples (A) and (B) are shown in Fig. 1. We found that the absorption peaks of sample (B) Tm^3+^(0.5%):telluride glass are positioned at 1699 nm, 1212 nm, 793 nm, 687 nm, and 465 nm. These absorption peaks are for the ^3^H_6_ → ^3^F_4_, ^3^H_6_ → ^3^H_5_, ^3^H_6_ → ^3^H_4_, ^3^H_6_ → ^3^F_3_, and ^3^H_6_ → ^1^G_4_ absorption transitions of the Tm^3+^ ions, respectively^16, 18^. We also found that the absorption peaks of the Er^3+^ ion of sample (A) Er(8.0%)Tm^3+^(0.5%):telluride glass are positioned at (1531 nm, 1497 nm), 977 nm, 795 nm, 653 nm, 544 nm, 522 nm, 489 nm, 451 nm, 408 nm, 380 nm, and 366 nm. These absorption peaks are for the ^4^I_15/2_ → ^4^I_13/2_, ^4^I_15/2_ → ^4^I_11/2_, ^4^I_15/2_ → ^4^I_9/2_, ^4^I_15/2_ → ^4^F_9/2_, ^4^I_15/2_ → ^4^S_3/2_, ^4^I_15/2_ → ^2^H_11/2_, ^4^I_15/2_ → ^4^F_7/2_, ^4^I_15/2_ → ^4^F_5/2_, ^4^I_15/2_ → ^2^H_9/2_, ^4^I_15/2_ → ^4^G_11/2_, and ^4^I_15/2_ → ^4^G_9/2_ absorption transitions of the Er^3+^ ions, respectively^16, 18^. The schematic diagram of the energy level structures for the Er^3+^ and Tm^3+^ ions are shown in Fig. 2.

### Excitation spectra

**Figure 3:**
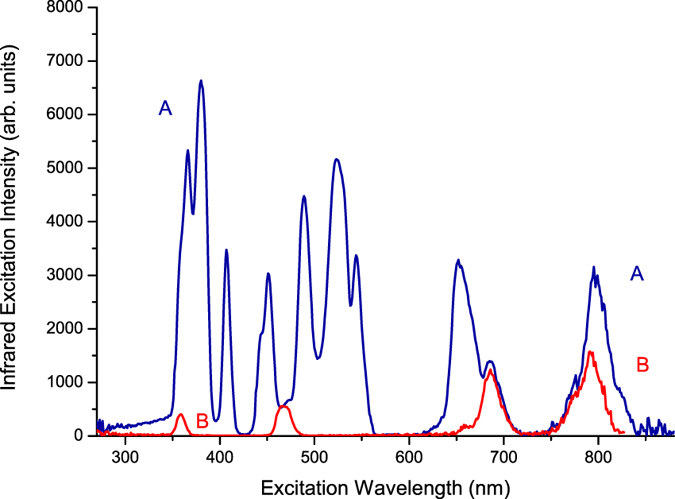
Infrared excitation spectra of samples (A) Er^3+^(8%)Tm^3+^(0.5%):telluride glass and (B) Tm^3+^(0.5%):telluride glass when monitored at 1800 nm for the ^3^F_4_ → ^3^H_6_ luminescence of the Tm^3+^ ions.

First, we selected the 1800-nm infrared luminescence wavelength of the Tm^3+^ ions in telluride glass to measure the infrared excitation spectra, from 250 nm to 850 nm, in sample (A) Er^3+^(8%)Tm^3+^(0.5%):telluride glass and sample (B) Tm^3+^(0.5%):telluride glass. The results are shown in Fig. 3. It was found that there are four excitation peaks for sample (B) Tm^3+^(0.5%):telluride glass, which are positioned at 358 nm, 467 nm, 686 nm, and 790 nm. Their excitation peak intensities are approximately 4.03 × 10^2^, 5.59 × 10^2^, 1.23 × 10^3^, and 1.56 ×  × 10^3^, respectively. These four excitation peaks are for the ^3^H_6_ → ^1^D_2_, ^3^H_6_ → ^1^G_4_, ^3^H_6_ → ^3^F_3_, and ^3^H_6_ → ^3^H_4_ transitions of the Tm^3+^ ion^16, 18^. It was also found that there are ten excitation peaks for sample (A) Er^3+^(8%)Tm^3+^(0.5%):telluride glass, which are positioned at 366 nm, 380 nm, 408 nm, 451 nm, 489 nm, 523 nm, 544 nm, 652 nm, 686 nm, and 795 nm. Their peak intensities are approximately 5.33 × 10^3^, 6.63 × 10^3^, 3.48 × 10^3^, 3.03 × 10^3^, 4.48 × 10^3^, 5.16 × 10^3^, 3.37 × 10^3^, 3.29 × 10^3^, 1.40 × 10^3^, and 3.16 × 10^3^, respectively. The excitation peaks at 366 nm, 380 nm, 408 nm, 451 nm, 489 nm, 523 nm, 544 nm, 652 nm, and 795 nm are for the ^4^I_15/2_ → ^4^G_9/2_, ^4^I_15/2_ → ^4^G_11/2_, ^4^I_15/2_ → ^2^H_9/2_, ^4^I_15/2_ → ^4^F_5/2_, ^4^I_15/2_ → ^4^F_7/2_, ^4^I_15/2_ → ^2^H_11/2_, ^4^I_15/2_ → ^4^S_3/2_, ^4^I_15/2_ → ^4^F_9/2_, and ^4^I_15/2_ → ^4^I_9/2_ transitions of the Er^3+^ ion, respectively^16, 18^. The excitation peak at 686 nm is for the ^3^H_6_ → ^3^F_3_ transition of the Tm^3+^ ions. The peak excitation intensity of 6.63 × 10^3^ for the 380 nm ^4^I_15/2_ → ^4^G_11/2_ transition of the Er^3+^ ions is, respectively, 16.5, 11.9, 5.4, and 4.3 times larger than the peak excitation intensities of 4.03 × 10^2^, 5.59 × 10^2^, 1.23 × 10^3^, and 1.56 × 10^3^ for the ^3^H_6_ → ^1^D_2_, ^3^H_6_ → ^1^G_4_, ^3^H_6_ → ^3^F_3_, and ^3^H_6_ → ^3^H_4_ transitions of the Tm^3+^ ions.

**Figure 4:**
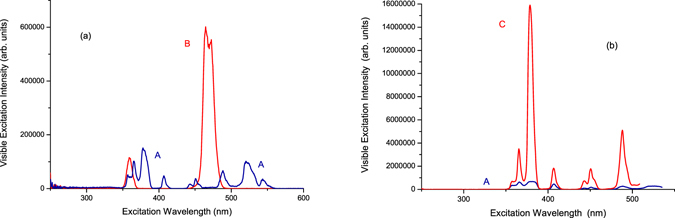
(**a**) The visible excitation spectra of samples (A) Er^3+^(8%)Tm^3+^(0.5%):telluride glass and (B) Tm^3+^(0.5%):telluride glass when monitored at 651 nm for the ^4^F_9/2_ → ^4^I_15/2_ luminescence of Er^3+^ ions and ^1^G_4_ → ^3^F_4_ luminescence of Tm^3+^ ions. (**b**) The visible excitation spectra of samples (A) Er^3+^(8%)Tm^3+^(0.5%):telluride glass and (C) Er^3+^(0.5%):telluride glass when monitored at 556 nm for the ^4^S_3/2_ → ^4^I_15/2_ luminescence of the Er^3+^ ions.

Then, we selected the 651-nm visible luminescence wavelength of Tm^3+^ ions in telluride glass to measure the visible excitation spectra, from 250 nm to 600 nm, in sample (A) Er^3+^(8%)Tm^3+^(0.5%):telluride glass and sample (B) Tm^3+^(0.5%):telluride glass. The results are shown in Fig. 4(a). From Fig. 4(a), the excitation spectrum of sample (B) Tm^3+^(0.5%):telluride glass, when monitored at the 651 nm visible luminescence wavelength, has two excitation peaks, which are positioned at 359.0 nm and 465.0 nm with peak intensities of 1.16 × 10^5^ and 6.02 × 10^5^, respectively. The 359.0 nm and 465.0 nm excitation peaks are attributed to the ^3^H_6_ → ^1^D_2_ and ^3^H_6_ → ^1^G_4_ transitions of the Tm^3+^ ions^16, 18^, respectively. However, the excitation spectrum of sample (A) Er^3+^(8%)Tm^3+^(0.5%):telluride glass, when monitored at the 651 nm visible luminescence wavelength, has approximately seven excitation peaks, which are positioned at 365.5 nm, 378.0 nm, 406.5 nm, 450.5 nm, 488.5 nm, 520.5 nm, and 544.5 nm with excitation peak intensities of 1.02 × 10^5^, 1.52 × 10^5^, 4.76 × 10^4^, 3.75 × 10^4^, 6.67 × 10^4^, 1.01 × 10^5^, and 3.15 × 10^4^, respectively. These excitation peaks correspond to the ^4^I_15/2_ → ^4^G_9/2_, ^4^I_15/2_ → ^4^G_11/2_, ^4^I_15/2_ → ^2^H_9/2_, ^4^I_15/2_ → ^4^F_5/2_, ^4^I_15/2_ → ^4^F_7/2_, ^4^I_15/2_ → ^2^H_11/2_, and ^4^I_15/2_ → ^4^S_3/2_ transitions of the Er^3+^ ions, respectively^16, 18^. This implies that the excitation energy for the 651 nm luminescence in sample (A) Er^3+^(8%)Tm^3+^(0.5%):telluride glass came from the Er^3+^ ions already. We also selected the 556 nm visible luminescence wavelength of Er^3+^ ions in the telluride glass to measure the visible excitation spectra, from 250 nm to 535 nm, in sample (A) Er^3+^(8%)Tm^3+^(0.5%):telluride glass and sample (C) Er^3+^(0.5%):telluride glass. These results are shown in Fig. 4(b). The spectrum of Fig. 4(b) is the characteristic excitation spectrum of Er^3+^ ions^16, 18^.

### Luminescence spectra

**Figure 5:**
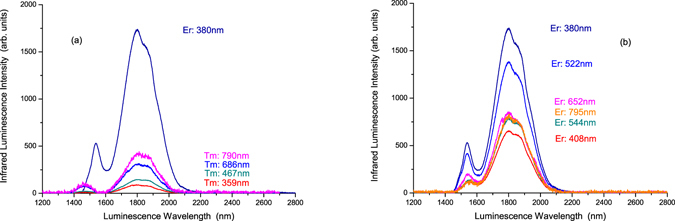
(**a**) Infrared luminescence spectra for sample (A) Er^3+^(8%)Tm^3+^(0.5%):telluride glass when excited by 380 nm, 358 nm, 467 nm, 686 nm, and 790 nm light for the ^4^I_15/2_ → ^4^G_11/2_ absorption of the Er^3+^ ions, ^3^H_6_ → ^1^D_2_, ^3^H_6_ → ^1^G_4_, ^3^H_6_ → ^3^F_3_, and ^3^H_6_ → ^3^H_4_ absorption of Tm^3+^ ions. (**b**) Infrared luminescence spectra for sample (A)Er^3+^(8%)Tm^3+^(0.5%):telluride glass *when* excited by 380 nm, 408 nm, 522 nm, 544 nm, 652 nm, and 795 nm light for the ^4^I_15/2_ → ^4^G_11/2_, ^4^I_15/2_ → ^2^H_9/2_, ^4^I_15/2_ → ^2^H_11/2_, ^4^I_15/2_ → ^4^S_3/2_, ^4^I_15/2_ → ^4^F_9/2_, ^4^I_15/2_ → ^4^I_9/2_ absorption of the Er^3+^ ions.

First, we selected the ^4^I_15/2_ → ^4^G_11/2_ absorption wavelength, 380 nm, of the Er^3+^ ions in sample (A) Er^3+^(8%)Tm^3+^(0.5%):telluride glass, and the ^3^H_6_ → ^1^D_2_, ^3^H_6_ → ^1^G_4_, ^3^H_6_ → ^3^F_3_, and ^3^H_6_ → ^3^H_4_ absorption wavelengths of 358 nm, 467 nm, 686 nm, and 790 nm of the Tm^3+^ ions in sample (B) Tm^3+^(0.5%):telluride glass as the excitation wavelengths to measure the infrared luminescence spectra at wavelengths from 1200 nm to 2800 nm. The results are shown in Fig. 5(a). There are two luminescence peaks for sample (A) Er^3+^(8%)Tm^3+^(0.5%):telluride glass, which are positioned at 1537 nm and 1800 nm. These luminescence peaks are the 1537 nm ^4^I_13/2_ → ^4^I_15/2_ luminescence of the Er^3+^ ions, and the 1800 nm ^3^F_4_ → ^3^H_6_ luminescence of the Tm^3+^ ions^16, 18^. The luminescence peak intensities of the 1537 nm and 1800 nm peaks are approximately 5.32 × 10^2^ and 1.73 × 10^3^, respectively. There are two luminescence peaks for sample (B) Tm^3+^(0.5%):telluride glass, which are positioned at 1468 nm and 1800 nm. These two luminescence peaks are for the 1468 nm ^3^H_4_ → ^3^F_4_ luminescence of the Tm^3+^ ions and the 1800 nm ^3^F_4_ → ^3^H_6_ luminescence of the Tm^3+^ ions^16, 18^. The luminescence peak intensities of the 1800 nm peaks, when the sample is excited by 358 nm, 467 nm, 686 nm, and 790 nm light, are approximately 8.90 × 10^1^, 1.45 × 10^2^, 3.10 × 10^2^, and 3.96 × 10^2^, respectively. The 1800-nm luminescence peak intensity, 1.73 × 10^3^, of sample (A) Er^3+^(8%)Tm^3+^(0.5%):telluride glass when excited by 380 nm light is approximately 19.5, 12.0, 5.6, and 4.4 times larger than that of sample (B) Tm^3+^(0.5%):telluride glass when excited by 358 nm, 467 nm, 686 nm, and 790 nm light, respectively.

We then selected the ^4^I_15/2_ → ^4^G_11/2_, ^4^I_15/2_ → ^2^H_9/2_, ^4^I_15/2_ → ^2^H_11/2_, ^4^I_15/2_ → ^4^S_3/2_, ^4^I_15/2_ → ^4^F_9/2_, and ^4^I_15/2_ → ^4^I_9/2_ absorption wavelengths of 380 nm, 408 nm, 522 nm, 544 nm, 652 nm, and 795 nm for the Er^3+^ ions in sample (A) Er^3+^(8%)Tm^3+^(0.5%):telluride glass as the excitation wavelengths to measure the infrared luminescence spectra, from 1200 nm to 2800 nm. The results are shown in Fig. 5(b). Their luminescence peak intensities are approximately 1.73 × 10^3^, 6.53 × 10^2^, 1.38 × 10^3^, 7.83 × 10^2^, 8.48 × 10^2^, and 8.17 × 10^2^, respectively.

**Figure 6:**
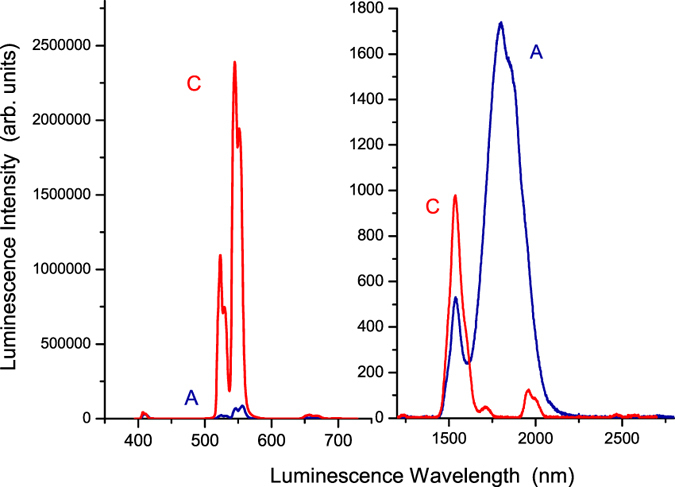
Visible and infrared luminescence spectra of samples (A) Er^3+^(8%)Tm^3+^(0.5%):telluride glass and (C) Er^3+^(0.5%):telluride glass when excited by 380 nm light for the ^4^I_15/2_ → ^4^G_11/2_ absorption of Er^3+^ ions.

In addition, we selected the ^4^I_15/2_ → ^4^G_11/2_ absorption wavelength, 380 nm, of the Er^3+^ ions as the excitation wavelength to measure the infrared luminescence spectra, from 1200 nm to 2800 nm, for sample (A) Er^3+^(8%)Tm^3+^(0.5%):telluride glass and sample (C) Er^3+^(0.5%):telluride glass. The results are shown in Fig. 6. There is only one main luminescence peak for sample (C) Er^3+^(0.5%):telluride glass, which is positioned at 1537 nm. This luminescence peak is the 1537 nm ^4^I_13/2_ → ^4^I_15/2_ transition of the Er^3+^ ions^16, 18^. Its luminescence peak intensity is approximately 9.78 × 10^2^. The ratio of the 1800-nm luminescence peak intensity of 1.73 × 10^3^ of sample (A) Er^3+^(8%)Tm^3+^(0.5%):telluride glass, to the 1537-nm luminescence peak intensity of 9.78 × 10^2^ of sample (C) Er^3+^(0.5%):telluride glass, is approximately 1.8. Meanwhile, the ratio of the 1800-nm luminescence integral area intensity of 4.76 × 10^5^ for sample (A) Er^3+^(8%)Tm^3+^(0.5%):telluride glass, to the 1537-nm luminescence integral area intensity of 9.55 × 10^4^ for sample (C) Er^3+^(0.5%):telluride glass, is approximately 5.0. From the results of Figs 5(a) and 6, we can conclude that the infrared luminescence intensity of sample (A) Er^3+^(8%)Tm^3+^(0.5%):telluride glass, is much larger than that of sample (B) Tm^3+^(0.5%):telluride glass or sample (C) Er^3+^(0.5%):telluride glass.

Finally, we selected the ^4^I_15/2_ → ^4^G_11/2_ absorption wavelength, 380 nm, of the Er^3+^ ions as the excitation wavelength to measure the visible luminescence spectra, from 395 nm to 728 nm, for sample (A) Er^3+^(8%)Tm^3+^(0.5%):telluride glass and sample (C) Er^3+^(0.5%):telluride glass. The results are also shown in Fig. 6. There are four luminescence peaks, which are positioned at 408.0 nm, 525.0 nm, (545.0 nm/556.0 nm), and 658.0 nm. These four luminescence peaks are for the ^2^H_9/2_ → ^4^I_15/2_, ^2^H_11/2_ → ^4^I_15/2_, ^4^S_3/2_ → ^4^I_15/2_, and ^4^F_9/2_ → ^4^I_15/2_ luminescence transitions of the Er^3+^ ions, respectively. Their luminescence peak intensities are approximately 3.56 × 10^4^, 2.25 × 10^4^, 8.62 × 10^4^, and 6.05 × 10^3^, respectively, for sample (A) Er^3+^(8%)Tm^3+^(0.5%):telluride glass, and are approximately 4.37 × 10^4^, 1.10 × 10^6^, 2.39 × 10^6^, and 2.58 × 10^4^, respectively, for sample (C) Er^3+^(0.5%):telluride glass.

### Lifetime dynamics

**Figure 7:**
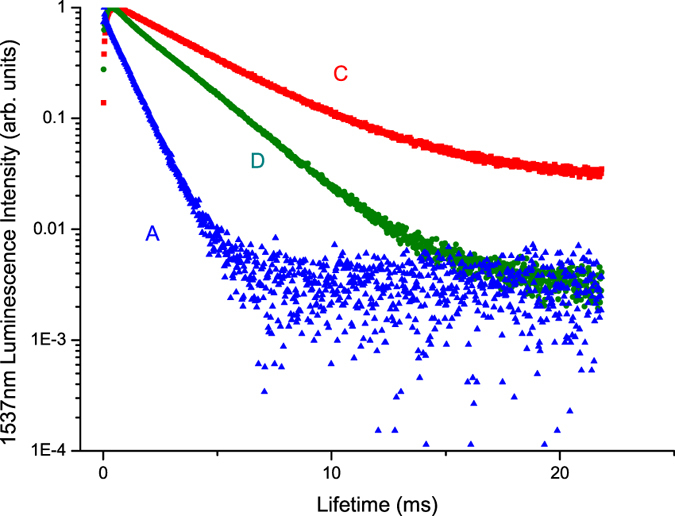
Lifetimes of the 1537-nm luminescence peaks in samples (A) Er^3+^(8%)Tm^3+^(0.5%):telluride glass, (C) Er^3+^(0.5%):telluride glass, and (D) Er^3+^(8.0%):telluride glass when excited by 380 nm light for the ^4^I_15/2_ → ^4^G_11/2_ absorption of Er^3+^ ions.

We used a 380-nm pulsed light from a xenon lamp as the excitation light source to measure the lifetimes of the 1537-nm luminescence peak in samples (A) Er^3+^(8%)Tm^3+^(0.5%):telluride glass, (C) Er^3+^(0.5%):telluride glass, and (D) Er^3+^(8.0%):telluride glass. The measured results are shown in Fig. 7.

According to the literature in the field of infrared quantum cutting, the efficiency of energy transfer among Er^3+^ ions can be calculated by using formula (1) below. Similarly, the efficiency of energy transfer between Er^3+^ and Tm^3+^ ions can be calculated by using formula (2)^1, 3, 5–12, 15, 20–28^:

1 $${\eta }_{tr,x \% Er}\approx 1-\frac{\int {I}_{x \% Er}dt}{\int {I}_{{\rm{0.5}} \% Er}dt}$$

2 $${\eta }_{tr,x \% Er,y \% Tm}\approx 1-\frac{\int {I}_{x \% Er,y \% Tm}dt}{\int {I}_{x \% Er}dt}$$

where I denotes the light intensity, *x*%Er represents the concentration of Er^3+^ ions, and y%Tm represents the concentration of Tm^3+^ ions. It is assumed that the energy transfer between Er^3+^ ions is negligible when *x* = 0.5%. Therefore, ${I}_{0.5 \% Er}$ can represent the case of non-energy transfer. It is known that there is an intense resonant energy diffusion {^4^I_13/2_(Er^3+^) → ^4^I_15/2_(Er^3+^), ^4^I_15/2_(Er^3+^) → ^4^I_13/2_(Er^3+^)} among Er^3+^ ions in (A) Er^3+^(8%)Tm^3+^(0.5%):telluride glass and (D) Er^3+^(*8*.0%):telluride glass, because both the concentrations of Er^3+^ ions and the populations in the ^4^I_13/2_(Er^3+^) first-excited state are very high. Therefore, resonant energy transfer to nearby Er^3+^ ions will be large. The excitations will lose their energy to impurity, defects, or trap states – a process referred to as concentration quenching. Formula (1) calculates only the efficiency of the resonant energy transfer.

From Fig. 7, we can calculate the integrated sum value for the luminescence lifetime curves of samples (A) Er^3+^(8%)Tm^3+^(0.5%):telluride glass, (C) Er^3+^(0.5%):telluride glass, and (D) Er^3+^(8.0%):telluride glass. The results are:$\int I{(1537nm)}_{0.5 \% Er}dt=4.886$, $\int I{(1537nm)}_{8.0 \% Er}dt=2.892$, and $\int I{(1537nm)}_{8 \% Er0.5 \% Tm}dt=0.874$. According to formula (1), we obtained the efficiency of resonant energy transfer {^4^I_13/2_ → ^4^I_15/2_, ^4^I_15/2_ → ^4^I_13/2_} among the Er^3+^ ions as follows: ${\eta }_{tr,8.0 \% Er}(1537nm)=40.8 \%$. From formula (2), we obtain the efficiency of energy transfer {^4^I_13/2_(Er^3+^) → ^4^I_15/2_(Er^3+^), ^3^H_6_(Tm^3+^) → ^3^F_4_(Tm^3+^)} between the Er^3+^ and Tm^3+^ ions as follows: ${\eta }_{tr,8 \% Er,0.5 \% Tm}=1-\frac{\int {I}_{8 \% Er,0.5 \% Tm}dt}{\int {I}_{8 \% Er}dt}=69.8 \%$.

**Figure 8:**
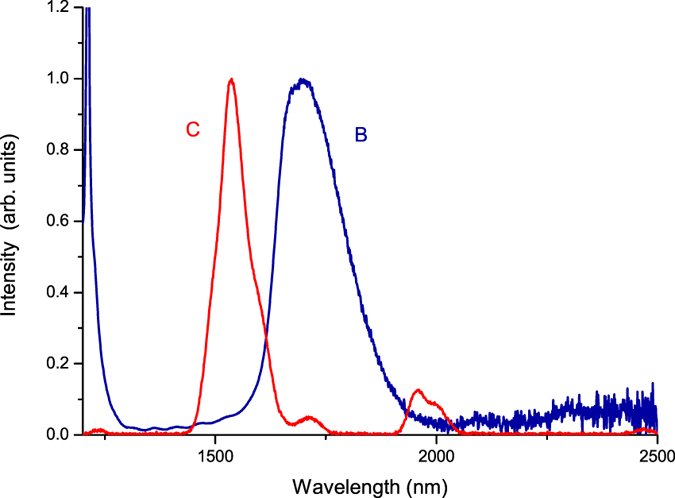
The infrared luminescence of sample (C) Er^3+^(0.5%):telluride glass when excited by 380-nm light for the ^4^I_15/2_ → ^4^G_11/2_ absorption of Er^3+^ ions and the infrared absorption of sample (B) Tm^3+^(0.5%):telluride glass.

Figure 8 shows the infrared luminescence of Er^3+^ ions and the infrared absorption of Tm^3+^ ions. We found that there is an obvious overlap between the infrared luminescence of Er^3+^ donor ions and the infrared absorption of Tm^3+^ acceptor ions. This results in a very strong energy transfer {^4^I_13/2_(Er^3+^) → ^4^I_15/2_(Er^3+^), ^3^H_6_(Tm^3+^) → ^3^F_4_(Tm^3+^)} between the Er^3+^ and Tm^3+^ ions. The transfer efficiency of ${\eta }_{tr,8 \% Er,0.5 \% Tm}=69.8 \%$ is very reasonable for photovoltaic applications.

## Discussion

From the results of the measurements shown in Figs 5 and 6 and their analyses, we found that the near-infrared 1800-nm luminescence intensity of sample (A) Er^3+^(8%)Tm^3+^(0.5%):telluride glass is approximately 4.4 to 19.5 times larger than that of sample (B) Tm^3+^(0.5%):telluride glass, and is approximately 5.0 times larger than that of sample (C) Er^3+^(0.5%):telluride glass. Meanwhile, from Fig. 6, the visible luminescence intensity of sample (A) Er^3+^(8%)Tm^3+^(0.5%):telluride glass is much smaller than that of sample (C) Er^3+^(0.5%):telluride glass. Moreover, from Figs 3 and 4, we found that the excitation spectra of the 1800-nm infrared luminescence and the 522 nm and 652 nm visible luminescences of sample (A) Er^3+^(8%)Tm^3+^(0.5%):telluride glass are very similar to those of the Er^3+^ ions of sample (C) Er^3+^(0.5%):telluride glass, with respect to the shape of the spectral waveforms and peak wavelengths. From Figs 7 and 8, we found an overlap and energy transfer between the infrared luminescence of the Er^3+^ donor ions and the infrared absorption of the Tm^3+^ acceptor ions. The efficiency of the first-order energy transfer {^4^I_13/2_(Er^3+^) → ^4^I_15/2_(Er^3+^), ^3^H_6_(Tm^3+^) → ^3^F_4_(Tm^3+^)} between the Er^3+^ and Tm^3+^ ions is approximately ${\eta }_{tr,8 \% Er,0.5 \% Tm}=69.8 \%$. From our previous work^28^, we know that a telluride glass with an 8% mol concentration of Er^3+^ ions will exhibit intense first-order near-infrared quantum cutting luminescence phenomena. It is obvious that there is no cross-energy transfer for samples (B) Tm^3+^(0.5%):telluride glass and (C) Er^3+^(0.5%):telluride glass, because their 0.5% concentration of rare earth ions is low. However, sample (A) Er^3+^(8%)Tm^3+^(0.5%):telluride glass has strong cross-energy transfer between Er^3+^ ions, because its 8% concentration of Er^3+^ ions is high. Therefore, we can conclude that the observed behaviour is an important multiphoton first-order near-infrared quantum cutting luminescence phenomenon of novel Er^3+^/Tm^3+^ ion pairs. Therefore, sample (A) Er^3+^(8%)Tm^3+^(0.5%):telluride glass first exhibits an intense first-order near-infrared quantum cutting among Er^3+^ ions, and then, the energy is transferred from Er^3+^ ions to Tm^3+^ ions. This results in the intense multiphoton first-order near-infrared quantum cutting 1800-nm luminescence of the Tm^3+^ ions.

The schematic diagrams of the energy-level structures of Er^3+^ and Tm^3+^ ions and the quantum cutting process are shown in Fig. 2.

When the ^4^G_11/2_ energy level is excited by 380-nm light, many Er^3+^ ions may populate at the ^4^G_11/2_ energy level because its absorption is very strong. The Er^3+^ ions undergo an intense {^4^G_11/2_  →  ^4^I_13/2_, ^4^I_15/2_ → ^2^H_11/2_} ET^r101^-ET^a06^ first-order cross-energy transfer process. The transition mismatch, ΔE = 623 cm^−1^, is moderate, but the reduced matrix elements (U^λ^)^2^ (0.1005, 0.2648, 0.2570) and (0.7158, 0.4138, 0.0927) of the Er^3+^ ions are very large^16, 18^, and the multiphonon non-radiative relaxation is moderate, therefore the first-order cross-energy transfer rate of {^4^G_11/2_ → ^4^I_13/2_, ^4^I_15/2_ → ^2^H_11/2_} ET^r101^-ET^a06^ is large. The population of the ^4^G_11/2_ energy level may be initially transferred to the first excited state ^4^I_13/2_ and the ^2^H_11/2_ energy levels mainly through the {^4^G_11/2_ → ^4^I_13/2_, ^4^I_15/2_ → ^2^H_11/2_} ET^r101^-ET^a06^ first-order cross-energy transfer process. The population in the ^2^H_11/2_ energy level may be sequentially transferred to ^4^I_13/2_ via {^2^H_11/2_ → ^4^I_9/2_, ^4^I_15/2_ → ^4^I_13/2_} ET^r63^-ET^a01^ and {^4^I_9/2_ → ^4^I_13/2_, ^4^I_15/2_ → ^4^I_13/2_} ET^r31^-ET^a01^. This would result in the intense four-photon first-order near-infrared quantum cutting of the ^4^I_13/2_ → ^4^I_15/2_ luminescence. Moreover, the ^3^F_4_ level of Tm^3+^ ions is positioned at a slightly lower energy than the ^4^I_13/2_ level of Er^3+^ ions. There is a significant overlap and a first-order energy transfer {^4^I_13/2_(Er^3+^) → ^4^I_15/2_(Er^3+^), ^3^H_6_(Tm^3+^) → ^3^F_4_(Tm^3+^)} between the Er^3+^ and Tm^3+^ ions. Furthermore, the back-energy transfer {^3^F_4_(Tm^3+^) → ^3^H_6_(Tm^3+^), ^4^I_15/2_(Er^3+^) → ^4^I_13/2_(Er^3+^)} might be relatively very small, since it is an anti-Stokes process. Therefore, this would result in the very intense multi-photon first-order quantum cutting 1800-nm luminescence of the Tm^3+^ ions. This is the main cross-energy transfer process, which is shown in the Fig. 2.

Meanwhile, there are the subordinate cross-energy transfer processes occurred between Er^3+^ and Tm^3+^ ions directly. For example, {^4^S_3/2_(Er^3+^) → ^4^I_9/2_(Er^3+^), ^3^H_6_(Tm^3+^) → ^3^F_4_(Tm^3+^)} first-order cross-energy transfer process is large also even the concentration of Er^3+^ and Tm^3+^ is 8% and 0.5%. Because its transition mismatch, ΔE = −82 cm^−1^, is small, its reduced matrix elements (U^λ^)2 (0, 0.0765, 0.2569) and (0.5375, 0.7261, 0.2382) of the Er^3+^ and Tm^3+^ ions are large^16, 18^, and its multiphonon non-radiative relaxation is small. The population of the ^4^S_3/2_ energy level may be initially transferred to the excited state ^4^I_9/2_(Er^3+^) and the ^3^F_4_(Tm^3+^) energy levels mainly through the {^4^S_3/2_(Er^3+^) → ^4^I_9/2_(Er^3+^), ^3^H_6_(Tm^3+^) → ^3^F_4_(Tm^3+^)} first-order cross-energy transfer process. The population in the ^4^I_9/2_(Er^3+^) energy level may be sequentially transferred to ^3^F_4_(Tm^3+^) via {^4^I_9/2_ → ^4^I_13/2_, ^4^I_15/2_ → ^4^I_13/2_} ET^r31^-ET^a01^ and {^4^I_13/2_(Er^3+^) → ^4^I_15/2_(Er^3+^), ^3^H_6_(Tm^3+^) → ^3^F_4_(Tm^3+^)}. This would result in the intense three-photon first-order near-infrared quantum cutting 1800-nm luminescence of the Tm^3+^ ions.

As we know, the energy band gap of GaN materials is approximately 3.4 eV, which corresponds to 27423 cm^−1^ (365 nm) light. It is easy to achieve very intense 380-nm luminescence in a GaN light emitting diode (LED)^26^. Therefore, using the excellent quantum cutting phenomenon in the novel Er^3+^/Tm^3+^ ion pair to construct a near-to-mid infrared (1.8–2.0 μm) laser pumped by a GaN LED is a significant and useful prospect^12, 31^. Quantum cutting, GaN LEDs, and near-to-mid infrared (1.8–2.0 μm) lasers are all currently hot topics in field of science and nature. It is possible to significantly enhance the properties of these lasers^12, 31^. To the best of our knowledge, the present manuscript is the first to report the first-order quantum cutting effect of an Er^3+^-Tm^3+^ ion pair.

To summarize, we measured the absorption, excitation, and luminescence spectra, as well as the lifetime dynamics of Er^3+^/Tm^3+^ co-doped telluride glasses. An interesting multiphoton near-infrared quantum cutting luminescence phenomenon from novel Er^3+^-Tm^3+^ ion pairs was found. This can facilitate the development of next-generation environmentally friendly germanium solar cells. In addition, using the excellent quantum cutting ability of our novel Er^3+^/Tm^3+^ ion pairs to construct a near-to-mid infrared (1.8–2.0 μm) laser pumped by GaN LEDs is a promising prospect.

## Methods

### Synthesis

The samples used in the present work were (A) Er^3+^(8%)Tm^3+^(0.5%):telluride glass, (B) Tm^3+^(0.5%):telluride glass, (C) Er^3+^(0.5%):telluride glass, and (D) Er^3+^(8.0%):telluride glass. The composition of sample (A) Er^3+^(8%)Tm^3+^(0.5%):telluride glass, for example, was 70TeO_2_–25ZnO-5La_2_O_3_–8Er_2_O_3_–0.5Tm_2_O_3_. The telluride glasses were manufactured using highly purified TeO_2_, ZnO, La_2_O_3_, Er_2_O_3_, and Tm_2_O_3_ powders as the starting materials. The well-mixed raw materials were placed in an alumina crucible. The samples were melted at 900 °C for 50 min under an oxygen atmosphere. A dry oxygen atmosphere was introduced to remove hydroxyl groups. The melts were then poured into a preheated stainless steel mould and annealed for several hours near the glass transition temperature, T_g_ (approximately 300 °C). The annealed samples were cut and polished to a size of 16 mm × 20 mm × 5.5 mm for optical measurements.

### Characterization

The equipment used in our experiment was a FL3-2iHR fluorescence spectrometer (Horiba-JY Co., America, Japan, and France). The excitation light source was a xenon lamp. The visible light detector was an R2658p photomultiplier. The infrared detector was a DSS-PS020T PbS detector. For all results, the signal intensities at the same wavelength in the same figure can be compared directly. The absorptions were measured using a UV3600 spectrophotometer (Shimadzu, Japan). The lifetime dynamics were recorded using the same fluorescence spectrometer, with an excitation wavelength of 378 nm, a measurement range of 22 ms, a peak present of 2.80 × 10^4^, a sweep present of 1.28 × 10^5^, and a delay of 0%.
